# Thermo- and Light-Responsive Polymer-Coated Magnetic Nanoparticles as Potential Drug Carriers

**DOI:** 10.3389/fbioe.2022.931830

**Published:** 2022-07-12

**Authors:** Guihua Cui, Hao Wang, Shengsen Long, Tianshuo Zhang, Xiaoyu Guo, Shuiying Chen, Toyoji Kakuchi, Qian Duan, Donghai Zhao

**Affiliations:** ^1^ Science and Technology Division, Jilin Medical University, Jilin, China; ^2^ Department of Materials Science and Engineering, Changchun University of Science and Technology, Jilin, China; ^3^ Jilin Vocational College of Industry and Technology, Jilin, China; ^4^ Division of Biotechnology and Macromolecular Chemistry, Graduate School of Engineering, Hokkaido University, Sapporo, Japan

**Keywords:** Poly(N-isopropylacrylamide) (PNIPAM), magnetic nanoparticles, thermo-sensitive, light-sensitive, Poly(6-[4-(4-methoxy phenyl azo)-phenoxyl- hexyl methacrylate]) (PAzoMA)

## Abstract

A series of thermo- and light-responsive copolymers of poly (*N*-isopropylacrylamide) (PNIPAM) and 6-[4-(4-methoxy phenyl azo)-phenoxyl-hexyl methacrylate) (AzoMA) (PNIPAM-b-PAzoMA) were synthesized *via* reversible addition–fragmentation chain transfer (RAFT) radical polymerization. The resulting copolymers had a narrow molecular weight distribution range of 1.06–1.24, in which *M*_n_ changed regularly with the monomer concentration. Subsequently, the diblock copolymers were successfully modified on the surface of iron oxide nanoparticles through the interaction between the chemical bonds to prepare Fe_3_O_4_@(PNIPAM-b-PAzoMA) nanoparticles. The size of fabricated nanoparticles with excellent thermo-sensitivity and photo-sensitivity was controlled at about 40–50 nm. Cell viability assays suggested that the nanoparticles showed no significant cytotoxicity and potential drug delivery in the tumor microenvironment.

## 1 Introduction

The cancer burden had risen to 19.3 million new cases and 10.0 million cancer deaths in 2020 (IARC, 2020). Cancer is characterized by highly metastatic and recurrent, traditional treatments such as surgery, chemotherapy, and radiation and is difficult to cure completely (Couzin-Frankel, 2013; Gotwals et al., 2017). Therefore, continuous endeavors by the researchers are being made to develop new low-cost and effective solutions for cancer treatments with few side effects, high specificity, and anti-metastasis (Lu et al., 2020; Baggiolini et al., 2021; Eberhardt et al., 2021). Polymeric nanomedicine has become crucial in clinical application with its precise and efficient targeting therapy of tumors (Farokhzad and Langer, 2006; Hawkins et al., 2008; Girase et al., 2019; Chen et al., 2021). A few drug-carrying polymeric nanoparticles (NPs) have been used in preclinical oncology treatment (Tong et al., 2012; Li et al., 2018; He et al., 2019). Stimulus-responsive NP drug delivery systems have attracted extensive attention because of their specificity, selectivity, and efficacy in tumor tissues and biocompatibility for normal cells (Karimi et al., 2016; Jia et al., 2018). The endogenous and exogenous (or external) stimuli could induce drug release from polymer vesicles. External stimulus-responsive factors included pH, magnetism, temperature, ultrasound, and light (Satarkar and Hilt, 2008; Brazel, 2009; Schroeder et al., 2009; Ge et al., 2012; Huo et al., 2014; Liu et al., 2014; Shanmugam et al., 2014; Olejniczak et al., 2015; Rwei et al., 2015; Alimoradi et al., 2016). Slight changes in the external environment could trigger the release of the polymer vesicles to the drug. The integration of multiple functions and various chemical nature of compounds to improve their functionalities was an innovative approach to obtaining nanoscale drug carriers.

Light-responsive nanocarriers have attracted extensive attention in the field of drug delivery (Olejniczak et al., 2015; Rwei et al., 2015). Using light as a trigger has many beneficial advantages. Light is easy to manipulate and make tiny adjustments. The photo-responsive nano-carrier acts as a biofriendly material, which could achieve accurate control of the location, time, and dose of released species (Sortino, 2012). The first example is azobenzene units, which could be handily transformed between *trans*- and *cis*-configuration to release encapsulated antineoplastic drugs. There are so many distinctions of azobenzene derivatives in physical, chemical, optical, and biological properties which are caused by *E*-to-*Z* isomerization. It is the reason that azobenzene compounds could be utilized to tune the characteristics of host materials. A growing number of studies were using azobenzene derivatives covalently linked to polymers as photoactivated drug delivery systems in NPs formed *via* self-assembly of polymers (Shibaev et al., 2003; Wang et al., 2015; Zhang et al., 2019; Shi et al., 2021a; Yao et al., 2021). It was reasonable to expect that as soon as the hydrophobic forces and *π*-*π* interactions are removed under UV irradiation, the vesicles with drugs dominated by electrostatic forces would expand rapidly to open the “gate” to achieve drug release (Yao et al., 2021).

Fe_3_O_4_ NPs are excellent nanoparticles for cancer therapy, which have been approved by the U.S. Food and Drug Administration (FDA) for the clinical treatment due to magnetic targeting and *T*_2_-weighted magnetic resonance imaging (MRI) (Ge et al., 2018). However, individual Fe_3_O_4_ NPs have no antitumor effect. Thus, composite materials based on ions have emerged, especially anti-tumor composites. In recent years, many substances have been used to combine with Fe_3_O_4_ to prepare antitumor nanoparticles, such as oxide-graphene (Lin et al., 2014), poly (ethylene glycol)-block-poly (lactic-co-glycolic acid) copolymer-encapsulated (Liu et al., 2022), and polymers (Swain et al., 2015; Du et al., 2017; Shi et al., 2021b; Ndebele et al., 2021). These composite Fe_3_O_4_ NPs showed great potential for cancer therapy. In the past few decades, the temperature sensitivity of poly (*N*-isopropylacrylamide) (PNIPAM) has been utilized to successfully synthesize so many drug nanocarriers with temperature responsiveness and high loading capacity. PNIPAM could respond to changes in the microenvironment temperature, which caused changes in the carrier structure and interactions with cells to achieve temperature-sensitive drug delivery and release (Liu et al., 2015; Bathfield et al., 2016; Nguyen et al., 2016). In our previous study, a series of biomaterials containing PNIPAM were also synthesized, and their lower critical solution temperature (LCST) was close to human body temperature (Cui G. et al., 2020; Cui G. H. et al., 2020). In this work, we took advantage of the thermo- and light-responsive properties of the polymer to synthesize polymer-coated magnetic nanoparticles, which may be used for drug delivery in the tumor microenvironment.

## 2 Materials and Methods

### 2.1 Materials and Instrumentation

*N*- isopropylacrylamide (Aldrich, 98%) was recrystallized twice from a hexane/benzene mixture (3/2, v/v). 2,2′-Azobis (2-methylpropionitrile) (AIBN) (J&K chemical, 98%), 1,3,5-trioxane (Sigma-Aldrich, 99%), 1-dodecanethiol (Sigma-Aldrich, ≥98%), tetrabutylammonium bromide (Sigma-Aldrich, 99%), carbon disulfide (Sigma-Aldrich, 99.9%), p-anisidine (Aladdin, 99%), 1,6-dibromohexane (Aladdin, 99%), phenol (Aladdin, 99%), methacrylic acid (J&K chemical, 98%), and MTT (3-[4,5-dimethylthiazol-2-yl]-2,5-diphenyltetrazolium were purchased from Acros. Human breast cancer cell (MCF-7) culture and NCTC clone 929 (L-929) were purchased from the Type Culture Collection of the Chinese Academy of Sciences, Shanghai.

The ^1^H nuclear magnetic resonance (NMR) spectra of polymers in CDCl_3_ were obtained on a Varian Unity 400 NMR spectrometer. The molecular weights (*M*_n_) and polydispersity (*M*_*w*_/*M*_n_) were measured by gel permeation chromatography (GPC) using a Waters 510 pump and a Model 410 differential refractometer at 25°C. The LCSTs of the polymer solutions were determined by turbidimetry, using a Shimadzu-2600 UV-Vis spectrophotometer with a heating rate of 0.1°C·min^−1^. FTIR spectra were recorded on a Shimadzu IR-8400S spectrometer. The average particle size and size distribution of the nanoparticles were characterized by dynamic light scattering (DLS) with a Malvern470 instrument at a fixed scattering angle of 90°, after being filtered by 0.45-μm Millipore filters. The scanning electron microscopy (SEM) images were obtained with JEOL JSM-6701SF. The morphologies of the nanoparticles were determined by transmission electron microscopy (TEM) with JEOL JEM-2100. Powder X-Ray Diffraction (XRD) was performed by using an X′-Pert PRO Philips X-ray diffractometer using Cu radiation (wavelength *λ* is 1.514056 $\overset{{}^{\circ}}{A}$ ) in the 2*θ* range of 10 ∼100° with a scan rate of 20°/min. X-ray photoelectron spectra (XPS) were examined on a PHI-5702 instrument using Mg Ka radiation with a pass energy of 29.35 eV. The optical density (OD) was measured at 570 nm with a microplate reader, model 550 (Bio-Rad, United States). Cell viability was determined as a percentage of the negative control (untreated cells).

### 2.2 General Procedure for the Thermo- and Light-Responsive Block Polymer Synthesis

#### 2.2.1 Synthesis of Dual-responsive Block Polymers

The procedure of AzoMA synthesis is shown in Supplementary Scheme S1. NaNO_2_ (3.0094 g, 43.6 mmol) was dissolved in deionized water (20 ml). Then, it was added to the hydrochloric acid solution of p-anisidine (5.0984 g and 41.4 mmol) solution and stirred at 0°C for 2 h. Miscible liquids were shifted to sodium hydroxide solution (4 g and 0.1 mol) of phenol (4.0902 g and 43.4 mmol) and stirred at 0°C for 2 h, and the pH was adjusted to neutral to obtain 4-hydroxy-4′-methoxy-azobenzene. The aforementioned product (2.43 g and 10 mmol) and 1, 6-dibromohexane (14.67 g and 53.6 mmol) and anhydrous K_2_CO_3_ (7.3922 g and 53.6 mmol) were added to acetone (100 ml) and stirred at 75°C for 24 h. The mixture was precipitated in petroleum ether (30∼60°C) and recrystallized by thermal filtration in ethyl acetate twice to obtain 1-bromo-6 -(4-methoxyazobenzene-4′-oxygen) hexane.

Methacrylic acid (0.2004 g, 2 mmol) and KHCO_3_(0.1002 g and 1 mmol) were dissolved in *N*,*N*-dimethylformamide(10 ml); then, 1-bromo-6-(4-methoxyazobenzene-4′-oxygen) hexane(0.3912 g, 1.09 mmol) was added dropwise under whisk and stirred at 65°C for 4 h. The pure AzoMA was acquired by column chromatography (the results are shown in Supplementary Figures S1, S2).

#### 2.2.2 Synthesis of PNIPAM-b-PAzoMA

**SCHEME 1:**
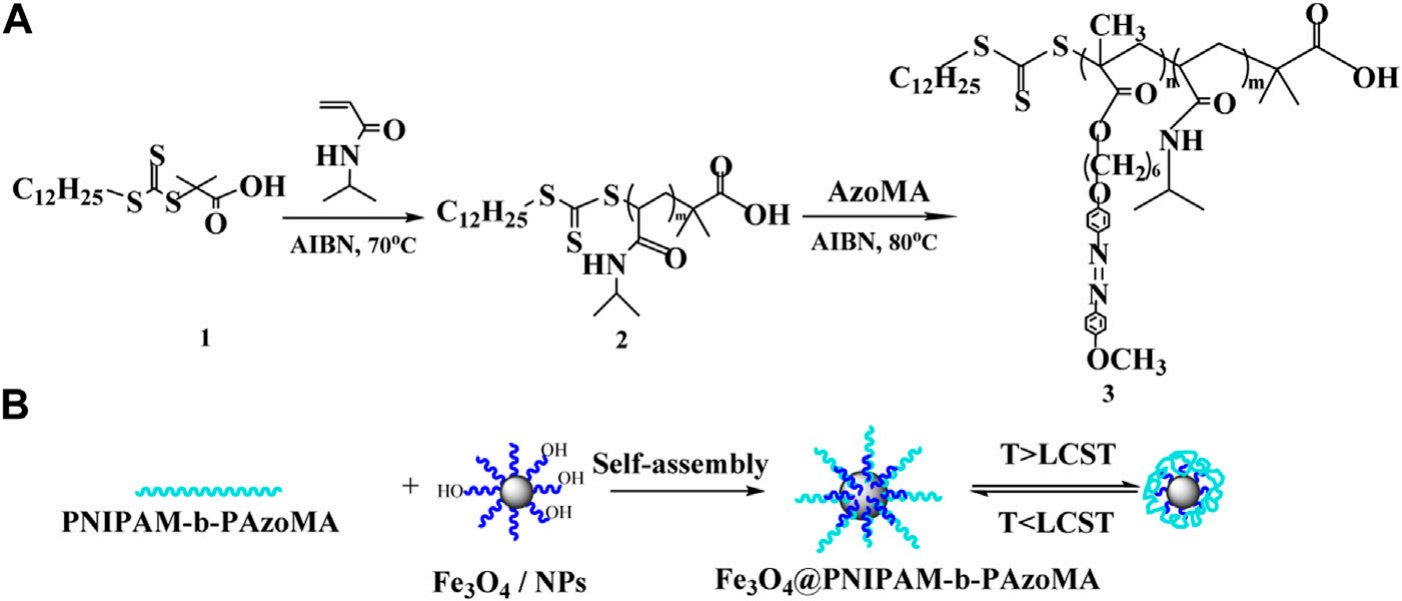
Fabrication strategy for thermo- and light-responsive polymer-coated magnetic nanoparticles.

PNIPAM-b-PAzoMA was synthesized using NIPAM and AzoMA as monomers by RAFT, as shown in Scheme 1. There were three steps for the synthesis of block copolymers. First, *S*-1-dodecyl-*S*´-(*α*,*α*′-dimethyl-*α*´´-acetic acid) trithio-carbonate (DMP) (1) was synthesized as a chain transfer agent (CTA). DMP was synthesized by a method derived from Lai et al. (2002), and we have reported in previous research (Cui G. et al., 2020). Subsequently, the synthesis of macromolecular chain transfer agents 2) *via* reversible addition–fragmentation chain transfer (RAFT) was mentioned in our study (Cui G. et al., 2020).

Finally, for the synthesis of PNIPAM-b-PAzoMA, a mixture of AzoMA (0.132 g and 0.5 mmol), macro CTA (152.7 mg and 0.015 mmol), 1,3,5-trioxane (50.44 mg and 0.56 mmol), and AIBN (1.70 mg and 0.01 mmol) was added to anhydrous DMF: H_2_O (7.5 ml and 95:5, v/v) and was sealed on the middle side of an H-shaped ampoule glass and stirred. Nitrogen was bubbled through both mixtures for 20 min to remove any oxygen. Three freeze-pump-thaw cycles were performed to degas the solutions. The ampoule was placed at 80°C for 24 h. Polymerization was quenched by exposing the solution to air. The solution was concentrated under a vacuum, and the polymer was precipitated into petroleum ether (30∼60°C) thrice; then, the product was dried under a vacuum.

### 2.3 Synthesis of Polymer-Coated Magnetic Nanoparticles

Fe_3_O_4_ nanoparticles were synthesized by chemical coprecipitation (Wu et al., 2012). The details are mentioned in our report (Cui G. H. et al., 2020). The Fe_3_O_4_ nanoparticles were dissolved in *N*, *N*-dimethylformamide (20 ml) and dispersed by ultrasonic concussion. Then PNIPAM-b-PAzoMA (0.3 mmol), EDC·HCl (10 mg and 0.052 mmol), and DMAP (7.1 mg and 0.058 mmol) were added successively under stirring and stirred at 80°C in an oil bath for 24 h. The compound was washed continuously with excess methanol in order to remove the unreacted polymer after magnetic separation and dried under vacuum to obtain polymer-coated magnetic nanoparticles.

### 2.4 Biocompatibility Study

The cell viabilities of macro-CTA, PNIPAM-b-PAzoMA, and Fe_3_O_4_@PNIPAM-b-PAzoMA nanoparticles were preliminarily investigated by using NCTC clone 929 (L-929) and human breast cancer cell (MCF-7) culture. Specific details were referred to in our previous studies (Cui G. H. et al., 2020). Six replicate wells were used for the control and test concentrations for each sample. The optical density was measured using a microplate reader at 492 nm. The cell viability (%) was calculated according to the following Eq. 1:

$$\text{Cell viability}\left(\text{\%}\right)=\left(A_{\text{sample}}/A_{\text{control}}\right)\times \mathrm{100\%,}$$ (1)

where *A*_sample_ was the absorbance of the cells incubated in DMEM and mixture, and *A*_control_ was the absorbance of the cells incubated in DMEM.

## 3 Results and Discussion

### 3.1 Architecture Analysis

**FIGURE 1:**
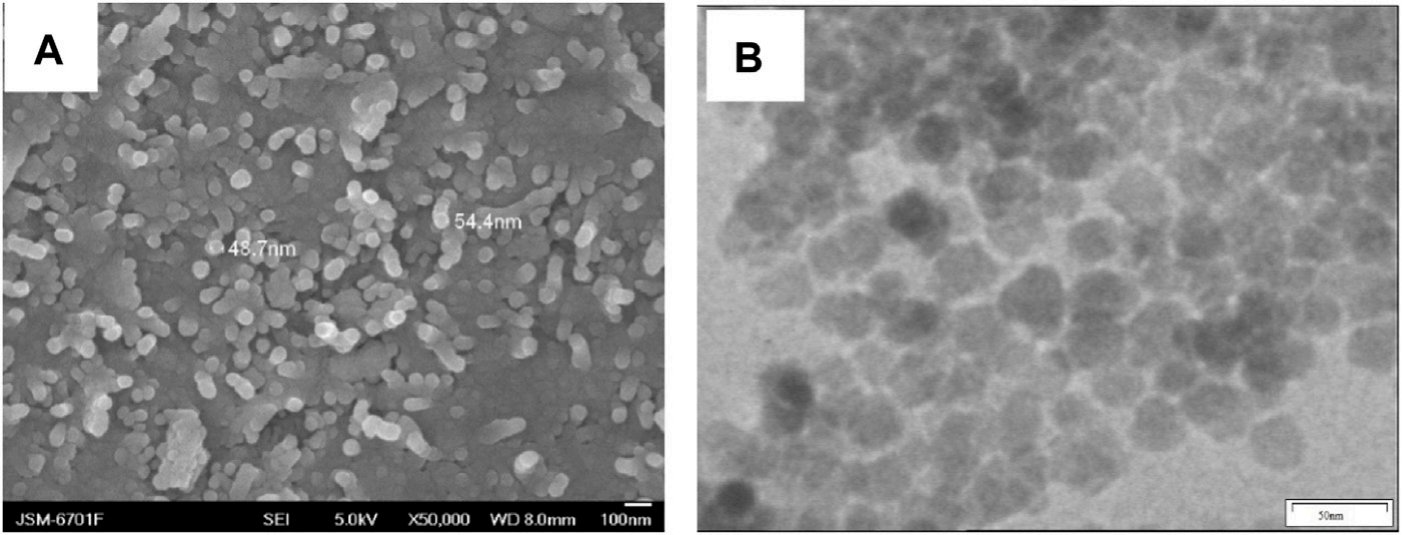
SEM and TEM images of Fe_3_O_4_@ PNIPAM-b-PAzoMA nanoparticles.

**FIGURE 2:**
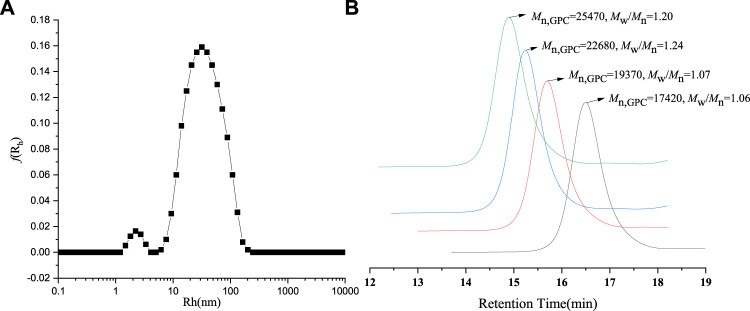
**(A)** DLS spectrogram for Fe_3_O_4_@ PNIPAM-b-PAzoMA nanoparticles. **(B)** GPC traces of PNIPAM-b-PAzoMA.

The morphologies of Fe_3_O_4_@PNIPAM-b-PAzoMA nanoparticles were investigated by SEM (Figure 1A) and TEM (Figure 1B). The diameter of the Fe_3_O_4_@PNIPAM-b-PAzoMA nanoparticles ranged from 40 to 50 nm. The size was basically identical to the intensity-average hydrodynamic radius distribution *f* (R_h_) which was measured by DLS in Figure 2A. The nanoparticles had an almost spherical shape since the polymer shells of Fe_3_O_4_@PNIPAM-b-PAzoMA nanoparticles were mostly formed by esterification.

The PNIPAM-b-PAzoMA was synthesized by the different feed ratios of AzoMA/macro-CTA/AIBN, which could obtain products that had a narrow molecular weight distribution range of 1.06–1.20, as shown in Figure 2B and Supplementary Table S2. The GPC traces of the block copolymer demonstrated a clean shift toward a lower elution volume. The macro-CTA was a polymer containing 132 PNIPAM units because it had the highest LCST among many macromolecular chain initiators prepared by our study.

**FIGURE 3:**
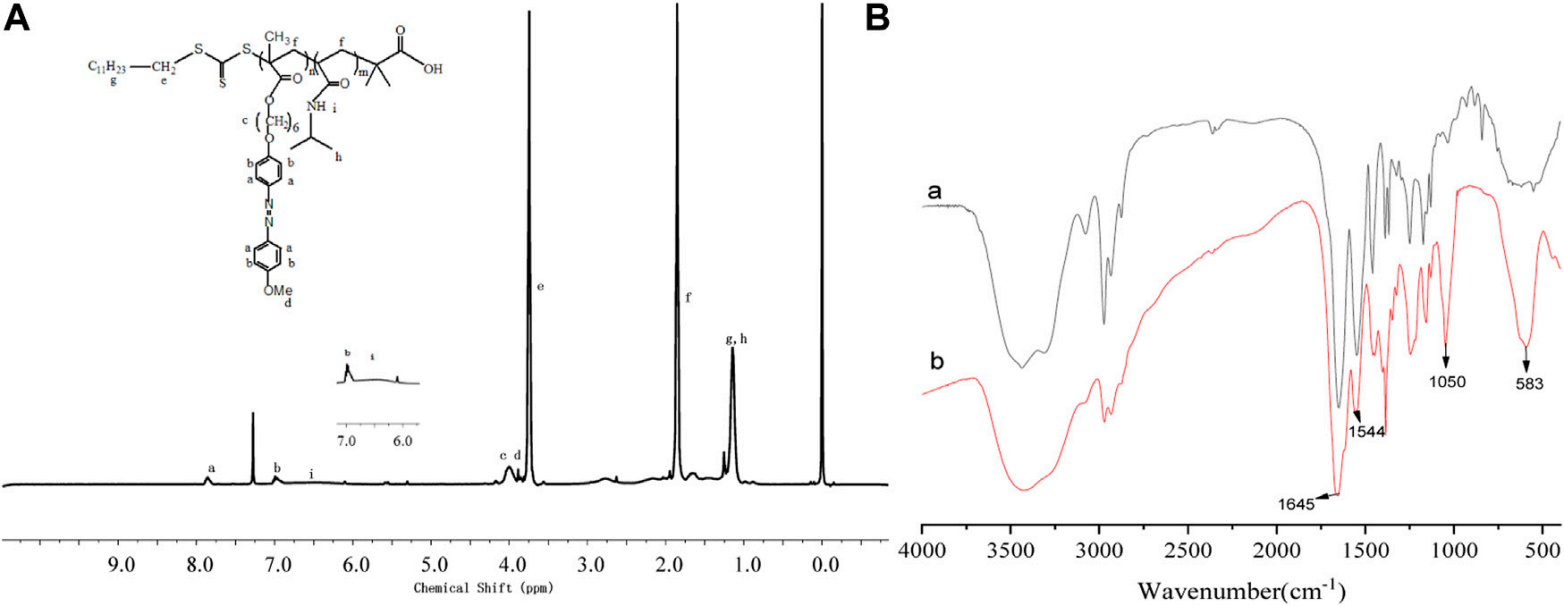
^1^HNMR spectra of PNIPAM-b-PAzoMA **(A)**, FT-IR spectra of PNIPAM-b-PAzoMA, and Fe_3_O_4_@PNIPAM-b-PAzoMA nanoparticles **(B)**.

The architecture of the PNIPAM-b-PAzoMA and Fe_3_O_4_@PNIPAM-b-PAzoMA nanoparticles was authenticated by FT-IR spectra and ^1^HNMR. From Figure 3A, compared with AzoMA (Supplementary Figure S1), a characteristic signal of PNIPAM representing nitrogen atoms was shifted to 6.53 ppm. The peaks at 7.85, 6.98, 3.99, and 3.89 ppm were the characteristic signals of AzoMA. It was seen clearly that the new characteristic peak of PNIPAM at 3,286 cm^−1^ was assigned to the stretching vibration (*ν*_N–H_) of the acylamino group in Figure 3B. The band at 1,645 fcm^−1^was ascribed to amide I [mainly the carbonyl stretching vibration (*ν*_C=O_)], and the band at 1,544cm^−1^ was ascribed to amide II [mainly the N–H bending vibration (*δ*_N–H_)], which has overridden the characteristic absorption peaks of AzoMA because of its high content of PNIPAM in the polymer. Two characteristic peaks appeared in the Fe_3_O_4_@PNIPAM-b-PAzoMA nanoparticles, which were located in 1,080 cm^−1^ and 583cm^−1^, and possessed the ester acid bond and Fe-O band.

**FIGURE 4:**
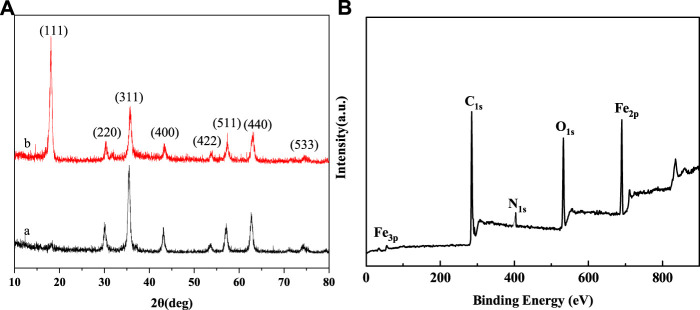
**(A)** XRD spectra of (a) bare Fe_3_O_4_ (b) Fe_3_O_4_@PNIPAM-b-PAzoMA nanoparticles and **(B)** XPS spectra of Fe_3_O_4_@PNIPAM-b-PAzoMA nanoparticles.

XPS and XRD spectra were used to further investigate the composition of the nanoparticles. Figure 4A showed the XRD pattern of the bare Fe_3_O_4_ and Fe_3_O_4_@PNIPAM-b-PAzoMA nanoparticles. The characteristic diffraction peaks for Fe_3_O_4_ (2*θ =* 18.07, 30.32, 35.68, 43.32, 53.78, 57.38, 62.89, and 74.56) marked by their indices on (111), (220), (311), (400), (422), (511), (440), and (533) crystal planes can be observed. All the peak positions were basically consistent with the standard data of the Fe_3_O_4_ structure (JCPDS card file No. 85–1,436) (Cullity, 1978). The peak position did no’t change, but the peak intensity changed differently. The dispersion of nanoparticles in the solution was different because of the effect of the surface polymer. In Figure 4B, peaks at the binding energies (BEs) of 284.39, 403, and 533.23eV were corresponding to C_1s_, N_1s_, and O_1s_, which were from the copolymer. The BE values of about 55.86eV and 688eV were ascribed to Fe_3p_ and Fe_2p_. These results indicated that the copolymer was successfully grafted onto the exterior surface of the Fe_3_O_4_ nanoparticles.

### 3.2 Thermo- and Light- Responsivity of Nanoparticles

**FIGURE 5:**
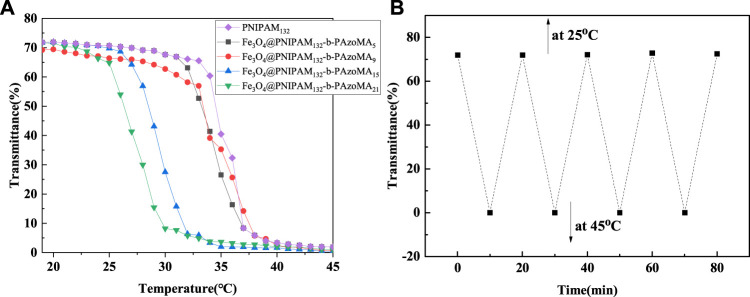
**(A)** Cloud point curve for the aqueous solution of macro-CTA and Fe_3_O_4_@PNIPAM-b-PAzoMA nanoparticles. **(B)** Plots of optical transmittance as a function of temperature for Fe_3_O_4_@(PNIPAM-b-PAzoMA) aqueous solution with the concentration of 1 mg/ml (10 min for each temperature four heating/cooling cycles between 25 and 45°C).

It was found that the LCST of macro-CTA was 33.9°C in Figure 5A. It was higher than the homopolymer PNIPAM due to the hydrophilic carboxyl group of the macro-CTA. The LCSTs of all Fe_3_O_4_@PNIPAM-b-PAzoMA nanoparticles were lower than those of macro-CTA. This might be caused by the hydrophobic interaction of AzoMA in the nanoparticles. Also, the content of AzoMA in nanoparticles was inversely proportional to LCST, as shown in Supplementary Figure S3.

The effect of AzoMA’s content on the LCST of the polymer was also reflected in the change of LCST of nanoparticles before and after UV irradiation, as shown in Table 1. The reason for this phenomenon was that the azo group of AzoMA was in a low dipole moment of the trans configuration without UV irradiation, and the polarity of AzoMA was weak, and this resulted in the strong hydrophobicity of AzoMA, and the LCST of the nanoparticles was lower than that of the macro-CTA. After UV irradiation, the azo group underwent a trans-cis transition. Compared with the trans configuration, the dipole moment of the azo group in the cis configuration was much higher, and the polarity of the AzoMA was also increased so that the hydrophilicity of nanoparticles was significantly enhanced. Another possible reason was that the azo of AzoMA in the trans configuration was a planar configuration. Compared with the cis structure, the superposition between molecules was more likely to occur so as to enhance the hydrophobic association ability, which was manifested by the increased phase transition temperature of the product after UV irradiation. After the aqueous solution of the sample irradiated by UV light was placed in the shade or exposed to visible light, its LCST returned to the temperature before UV irradiation. This was due to a cis-trans configuration transition of the azo group in Figure 5B.

**TABLE 1:** Summarized data on Fe3O4@PNIPAM-b-PAzoMA.

Sample	Amount of AzoMA (mol%)	LCST (%) before irradiation (oC)	LCST after irradiation (oC)	Δ (°C)LCST (oC)
F (°C)e3O4@(PNIPAM132-b-PAzoMA5)	3.6	31.5	31.8	0.3
Fe3O4@(PNIPAM132-b-PAzoMA9)	6.4	30	30.9	0.9
Fe3O4@(PNIPAM132-b-PAzoMA15)	10.2	26.9	29.8	2.9
Fe3O4@(PNIPAM132-b-PAzoMA21)	13.7	25	29	4

**FIGURE 6:**
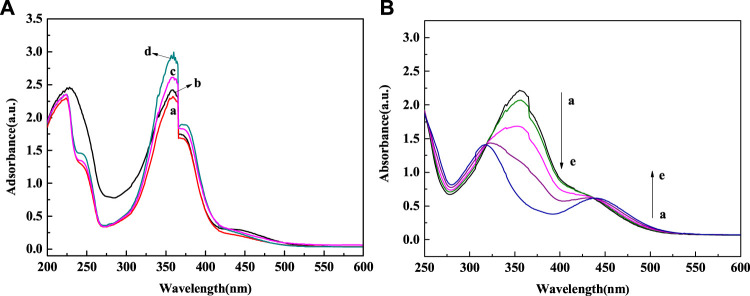
**(A)** Spectral changes of Fe_3_O_4_@PNIPAM-b-PAzoMA in aqueous solution during UV irradiation (365 nm, 8 W) (a) Fe_3_O_4_@PNIPAM_132_-b-PAzoMA_5_, (b) Fe_3_O_4_@PNIPAM_132_-b-PAzoMA_9_, (c) Fe_3_O_4_@PNIPAM_132_-b-PAzoMA_15_, and (d)Fe_3_O_4_@PNIPAM_132_-b-PAzoMA_21_. **(B)** Spectral changes of Fe_3_O_4_@PNIPAM_132_-b-PAzoMA_21_ under irradiation time: (a) 0 min, (b) 5 min, (c) 10 min, (d) 15 min, and (e) 20 min.

Figure 6 showed the UV spectrum changes of Fe_3_O_4_@PNIPAM-b-PAzoMA aqueous solutions with different contents of AzoMA that were irradiated by UV light. In the Figure 6A, the absorbance was proportional to the content of AzoMA. The sample showed a strong absorption peak at 358 nm, while the absorption peak at 450 nm was relatively weak. This was because AzoMA mainly with its trans configuration under such conditions, and the trans configuration of azobenzene was relatively low. Therefore, this study focused on Fe_3_O_4_@PNIPAM-b-PAzoMA nanoparticles with high AzoMA contents. However, in Figure 6B, the characteristic absorption peak of AzoMA’s cis configuration at 450 nm increased with the extension of UV irradiation time. This was because the azo groups in AzoMA gradually changed from trans configuration to cis configuration with the extension of UV irradiation time.

### 3.3 Thermostability and Magnetism of Nanoparticles

**FIGURE 7:**
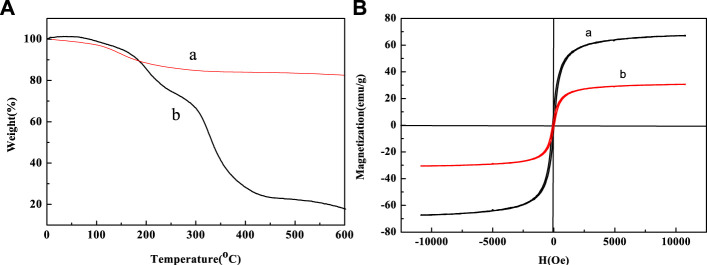
**(A)** TG curves of (a) Fe_3_O_4_ and (b) Fe_3_O_4_@PNIPAM-b-PAzoMA and **(B)** hysteresis loops of (a)bare Fe_3_O_4_ nanoparticles and (b)Fe_3_O_4_@PNIPAM-b-PAzoMA nanoparticles at room temperature.

The thermogravimetric analysis (TG) is shown in Figure 7. We could clearly determine that below 600°C, after grafting PNIPAM-b-PAzoMA onto the surface of the Fe_3_O_4_@PNIPAM-b-PAzoMA nanoparticles, the weight loss was about 56%, while only about 7% of weight loss for bare Fe_3_O_4_ nanoparticles. Compared with bare Fe_3_O_4_ nanoparticles, the weight loss of Fe_3_O_4_@PNIPAM-b-PAzoMA is generally concentrated in three stages which were 70°C∼150°C, 150°C∼300, and 300°C∼500°C. In the first phase, the quality loss was about 5%, which was roughly consistent with the loss in 1), and this value was identified as the loss of Fe_3_O_4_. The mass loss in the second phase was mainly due to the thermal decomposition of PAzoMA. The weight loss in the third period was relatively large, especially due to the thermal decomposition of PNIPAM in the polymer, which was basically consistent with the data measured by GPC.

Figure 7B shows the hysteresis graph of Fe_3_O_4_ and Fe_3_O_4_@PNIPAM-b-PAzoMA nanoparticles. Due to the small particle size, the saturation magnetization (Ms) of Fe_3_O_4_ was about 68.02 emu/g. The Ms of Fe_3_O_4_@PNIPAM-b-PAzoMA nanoparticles decreased significantly, which was 30.01 emu/g. This was because polymer-coated Fe_3_O_4_ changed the magnetic anisotropy and increased the directional obstruction on the surface. Fe_3_O_4_@PNIPAM-b-PAzoMA nanoparticles showed no coercivity, so we could judge that the nanoparticles had a certain degree of super-paramagnetism.

### 3.4 Assessment of the Cell Viability

**FIGURE 8:**
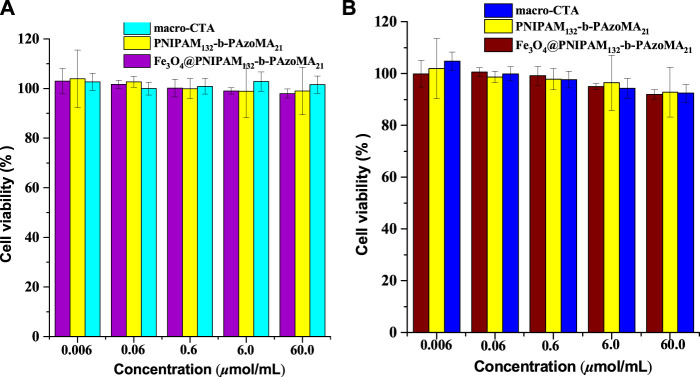
Cell viability of **(A)** the L-929 cells and **(B)** the MCF-7 cells incubated with the samples (macro-CTA, PNIPAM-b-PAzoMA, and Fe_3_O_4_@PNIPAM-b-PAzoMA nanoparticles), over a range of sample concentrations from 0.006 to 60 μmol/ml by MTT assay for 48 h.

The cytotoxicity study on normal cells (L-929, with high activity) and tumor cells (MCF-7, with insensitive to apoptosis induced by various chemotherapeutic drugs) was carried out to investigate the preliminary biocompatibility of the resulting Fe_3_O_4_@PNIPAM-b-PAzoMA nanoparticles in Figure 8. MTT studies showed that macro-CTA, PNIPAM-b-PAzoMA, and Fe_3_O_4_@PNIPAM-b-PAzoMA nanoparticles had very low toxicity to L-929 cells and MCF-7 cells, and the cell survival rate remained above 80% even at sample concentrations up to 60 μmol/ml.

## 4 Conclusion

In this study, the thermo- and light-responsive polymer-coated magnetic nanoparticles were synthesized. The size of the Fe_3_O_4_@PNIPAM-b-PAzoMA nanoparticles was about 40–50 nm. Fe_3_O_4_@PNIPAM-b-PAzoMA nanoparticles had excellent temperature sensitivity, photosensitivity, thermostability, and super-paramagnetism and no significant cytotoxicity. Fe_3_O_4_@PNIPAM-b-PAzoMA may be used for good drug delivery in the tumor microenvironment and for potential antitumor therapy.

## Data Availability

The original contributions presented in the study are included in the article/Supplementary Material; further inquiries can be directed to the corresponding authors.
