# Supplementary material for: Thermo- and Light-Responsive Polymer-Coated Magnetic Nanoparticles as Potential Drug Carriers
Source: Front Bioeng Biotechnol. 2022 Jul 12;10:931830. doi: 10.3389/fbioe.2022.931830 (PMC9315361; doi:10.3389/fbioe.2022.931830)
Supplement: Supplementary file 1 [file DataSheet1.docx]

**Synthesis of thermo- and light-responsive polymer-coated magnetic nanoparticles as a potential drug delivery in tumor microenvironment**

Guihua Cui^a,b^, Hao Wang ^a^^†^, Shengsen long^a^, Tianshuo Zhang^a^, Xiaoyu Guo^c^, Shuiying Chen^b^, Toyoji Kakuchi^d^, Qian Duan^*b^, Donghai Zhao*^a^

^a^ Science &Technology Division, Jilin Medical University, Jilin, Jilin, 132013, China.

^b^ Department of Materials Science and Engineering, Changchun University of Science and Technology, Changchun, Jilin, 130022, China.

^c^Jilin Vocational College of Industry and Technology, Jilin, Jilin, 132013, China.

^d^Division of Biotechnology and Macromolecular Chemistry, Graduate School of Engineering, Hokkaido University, Sapporo, 060-8628, Japan.

^†^These authors have contributed equally to this work

E-mail: [duanqian88@hotmail.com](mailto:duanqian88@hotmail.com), [cuiyuhan1981_0@sohu.com](mailto:cuiyuhan1981_0@sohu.com); Fax: +86 431 85306769, +86 432 4560117; Tel: +86 431 85583105, +86 432 64560187

**The** **procedure of AzoMA was shown in Scheme S1.**

Scheme S1 The fabrication procedure for AzoMA.

**Analysis of AzoMA**

The characteristic signals of benzene were showed in Fig.S1 at 7.86 ppm and 6.99ppm. Two other distinct chemical shifts at 6.09 ppm and 5.55 ppm were attributed to the hexyl acrylate group. The peaks at 4.16 ppm and 4.03ppm were derived from the methylene that's attached to the oxygen atom of AzoMA. And the shift of protons in methoxy group was 3.87ppm.


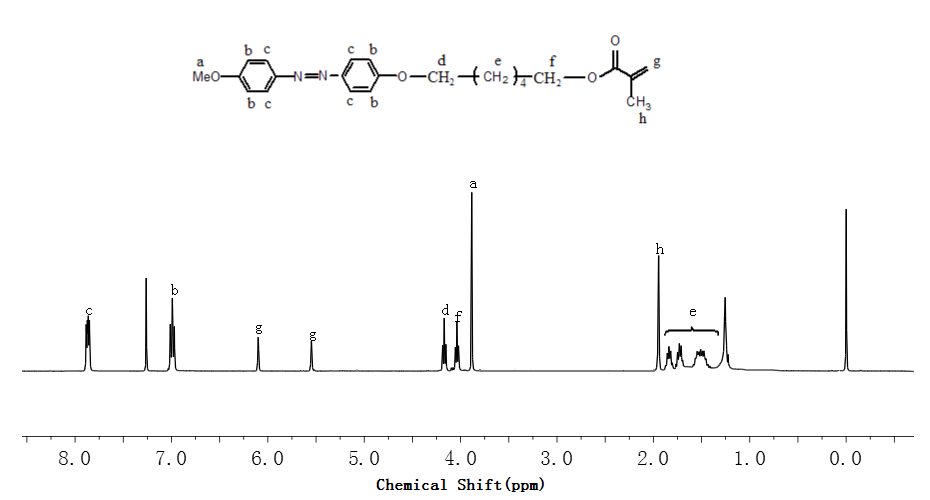


Fig. S1 ^1^H NMR spectra of the AzoMA in CDCl_3_.

The FT-IR spectra of the AzoMA was showed in Fig.S2. The stretching vibration peak of azo groups (1600cm^-1^and 1585cm^-1^) were showed in Fig. S2(a) and the 1704cm^-1^ was characteristic peak of ester group in Fig. S2(c). The characteristic peak atmospheres of methyl and methylene are located at 2942cm^-1^and 2862cm^-1^. The peak at 1637cm^-1^ was the carbon-carbon double bonds of AzoMA.

Fig.S2 FT-IR spectra of the (a)HEAB,(b)BrEAB and (c)AzoMA

**Analysis of PNIPAM-b-PAzoMA**

Table S1 showed the polymerization data of PNIPAM-b-PAzoMA.

Table S1 The polymerization data of macro-PNIPAM

polymer *M*_n,theo_^a^(g/mol) *M*_n, GPC_^b^ *M*_w_/*M*_n_^b^

PNIPAM_53_ 6380 6360 1.04

PNIPAM_84_ 9880 9800 1.05

PNIPAM_104_ 12140 12100 1.14

PNIPAM_132_ 15300 15310 1.13

Table S2 The polymerization data of PNIPAM-b-PAzoMA

polymer AzoMA/[AIBN]/ Time(h) *M*_n,theo_^a^(g/mol) *M*_n, GPC_^b^ *M*_w_/*M*_n_^b^

[macro-CTA]

PNIPAM_132_-b-PAzoMA_5_ 25/0.1/1 24 17830 17420 1.06

PNIPAM_132_-b-PAzoMA_9_ 50/0.1/1 24 19850 19370 1.07

PNIPAM_132_-b-PAzoMA_15_ 75/0.1/1 24 22890 22680 1.24

PNIPAM_132_-b-PAzoMA_21_ 100/0.1/1 24 25920 25470 1.20

**Thermo- and light- responsivity of Fe_3_O_4_@PNIPAM-b-PAzoMA nanoparticles**

Fig. S3 The relationship between LCST and content of AzoMA in Fe_3_O_4_@(PNIPAM-b-PAzoMA) during UV irradiation: (a) before irradiation, (b) after irradiation
